# Who Likes Sweets? Sweet Patterns: Influence of Sex, Age, Body Mass Index, Smoking and Olfactory Efficiency on the Consumption of Sweet Products

**DOI:** 10.3390/nu17213487

**Published:** 2025-11-06

**Authors:** Agata Lebiedowska, Magdalena Kamińska, Beata Krusiec-Świdergoł, Barbara Błońska-Fajfrowska, Magdalena Hartman-Petrycka

**Affiliations:** Department of Basic Biomedical Science, Faculty of Pharmaceutical Sciences in Sosnowiec, Medical University of Silesia, 40-055 Katowice, Polandmhartman@sum.edu.pl (M.H.-P.)

**Keywords:** food preferences, sweet products, fruits, sweet drinks, sex, age, body mass index, smoking and smell

## Abstract

**Background:** Sugars, also known as saccharides or carbohydrates, are essential organic compounds that ensure the human body functions properly. They are used as sources of energy, as structural elements and reserve materials. Excessive sugar consumption is prevalent in many countries and has negative health consequences. **Methods:** A total of 283 people living in Poland took part in the study. An interview and olfactory tests (dynamic olfactometry method) were conducted together with assessments of food preferences from 25 types of food products. **Aim:** To assess the impact of olfactory efficiency and individual characteristics, such as sex, age, body weight and tobacco addiction, on preferences for various sweet products. Another important objective of this study was to examine the patterns in preferences for different sweet foods. **Result and Conclusions:** Of all the types of sweet products analysed in the study, desserts and fruit were the most popular. Preferences varied depending on the type of sweet food or drink as well as on other factors. In terms of declared enjoyment, desserts, chocolate and sweets, including jellies and bread, formed a common group (Factor Three: ‘sweet products’); while sweet, carbonated drinks formed the group—Factor One (‘junk food’); and fruit, together with vegetables, salads, cheese and spicy dishes, formed the group—Factor Four. The most important factors influencing the preference for sweet foods and drinks were the severity of tobacco addiction, age and sex. There was no significant influence from olfactory efficiency or body mass index on the preference for any of the sweet products in the study.

## 1. Introduction

Sugars, also known as saccharides or carbohydrates, are a group of essential organic compounds that ensure the proper functioning of the human body. They differ in their chemical structure, physicochemical properties and susceptibility to digestion in the human digestive tract. These compounds are used as sources of energy, structural elements and reserve material [1,2].

Carbohydrates are divided into categories according to their structure: simple carbohydrates—monosaccharides (such as glucose, fructose and galactose); and complex carbohydrates, including disaccharides (such as sucrose and lactose), oligosaccharides (such as maltodextrins and galactosides), and polysaccharides (such as starch, cellulose and pectins). Disaccharides consist of two monosaccharide molecules linked by a glycosidic bond. Sucrose consists of glucose and fructose, while lactose consists of glucose and galactose. Polysaccharides, on the other hand, are macromolecular polymers composed of monosaccharide units linked by glycosidic bonds in straight or branched chains (e.g., starch, glycogen, cellulose and pectins) [1,2,3,4].

From a nutritional point of view, carbohydrates are categorised as either digestible or non-digestible. Metabolizablecarbohydrates are broken down and absorbed in the small intestine, where they are involved in the body’s metabolic processes. The main types of digestible carbohydrate are monosaccharides (such as glucose and fructose), disaccharides (such as sucrose and lactose), maltooligosaccharides, and starch [5,6]. There are two forms of starch: rapidly digested starch, which is digested within 20 min of consumption and causes a rapid increase in blood glucose concentration; and slowly digested starch, which takes 20–120 min to digest and causes a slower glycaemic effect. Non-digestible carbohydrates, however, are compounds that are neither digested nor absorbed in the small intestine. They are partially hydrolysed only in the colon through the action of intestinal microflora. This group includes cellulose, hemicellulose, pectin, resistant oligosaccharides, and resistant starch—all components of dietary fibre [1,5,6,7].

One of the major current public health problems is the excessive supply of carbohydrates, particularly simple sugars and sucrose. These substances are found in significant amounts in sweets, confectionery, fruit drinks, lemonades, nectars, colas, energy drinks, isotonic drinks and flavoured waters [8,9,10]. Consuming some of these products can equate to consuming several, maybe even a dozen or so, teaspoons of sugar or its substitutes per day. Simple sugars are rapidly metabolised, resulting in a sharp increase in postprandial glycaemia and an osmotic effect in the digestive system [5,11]. In recent years, scientific literature has emphasised the adverse effects of excessive fructose consumption on human metabolism. Excess fructose in the diet promotes disorders of lipid metabolism, including hyperlipidaemia and hypercholesterolaemia, as well as morning and postprandial increases in triglyceride levels. The metabolism of fructose produces significant amounts of 3-phosphoglyceric aldehyde, a precursor for the synthesis of fatty acids that determines its strong lipogenic effect [9,12,13]. Excessive sugar intake also contributes to overweight and obesity, insulin resistance, type 2 diabetes, metabolic syndrome, certain cancers, non-alcoholic fatty liver disease, and dental caries [8,14,15,16].

Given the key role of carbohydrates in the proper functioning of the human body, numerous studies are being conducted to assess the relationship between their molecular structure and perceived taste. These studies cover both receptor-dependent and receptor-independent pathways [3]. There are also reports indicating that the taste reception and perception of sweetness changes depending on the body’s metabolic state, which calls into question the universality of the preference for sweet foods, i.e., whether people who like sweet foods have different phenotypes from those who do not [17,18].

Food preferences, including preferences for sweet products, have been shown to be influenced by individual characteristics. Sex differences in sweet taste preferences have been documented [19], with women generally reporting higher preferences for sweet foods and chocolate compared to men. These differences may be related to hormonal factors, cultural influences, and variations in taste receptor sensitivity [20]. Age significantly affects taste perception, with children and adolescents preferring higher sweetness levels, while sensitivity to sweet tastes decreases in older adults [21]. This age-related decline in sweet taste sensitivity may influence food choices and dietary patterns throughout the lifespan, potentially affecting nutritional status in elderly populations. The relationship between body weight, BMI, and sweet preferences is complex and bidirectional [19]. Some studies suggest that higher BMI is associated with altered taste perception and increased preferences for energy-dense sweet foods, though the causal direction of this relationship remains debated [19]. Smoking significantly alters taste perception, with smokers showing reduced sensitivity to sweet tastes and lower hedonic ratings for sweet foods [22,23]. This impairment in taste function may persist for weeks to months after smoking cessation, affecting dietary choices during and after the quitting process. Finally, olfactory function is closely linked to taste perception and may influence food preferences [24]. Given that aroma contributes significantly to overall flavour perception, individuals with reduced olfactory function may experience altered food preferences and reduced enjoyment of foods. However, the specific role of olfactory efficiency in sweet product preferences has been less extensively studied, representing an important gap in current knowledge. In this regard, it is reasonable to assess the impact of olfactory sensitivity, as well as other individual characteristics such as sex, age, body weight, and tobacco addiction, on preferences for various sweet products. Another important objective of this study is to examine patterns of preference for different types of sweet food, a topic that has rarely been analysed in the existing literature. We hypothesised that preferences for sweet products would be associated with olfactory efficiency and moderated by sex, age, BMI, and smoking habits.

## 2. Materials and Methods

The results presented in this paper are part of a larger research project. The first part of the results, analysing the effect of different influences on the food preferences for products in Factor One group—Unhealthy Foods with a strong flavour—has already been published [25]. The second part of the results assessed the impact of various factors on food preferences for products in the second group: ‘Factor Two—Meat, Fish and Seafood’, which has also been published [26]. This study analyses the effect of the same influences on the next food product group: Factor Three—‘sweet products’. As the source of data is from the same project as in the publications on Unhealthy Foods and Meat, Fish and Seafood, the chapter on Materials and Methods and some of the results are identical to the first and second publication in this series [25,26].

### 2.1. Participants

The study involved 283 people aged between 18 and 82, living in the Silesian Voivodeship in Poland. The majority of the young people participating in the study were recruited among students of medical analysis, pharmacy, cosmetology and biotechnology of the Medical University of Silesia. As the research was conducted within the Students’ Science Club, a significant number of the participants were the students’ family and friends. There were 190 women and 93 men, including 43 smokers, who participated in the study. Participants self-reported their biological sex (male/female) during the initial questionnaire. The percentage of people belonging to each body mass index (BMI) category, according to WHO (World Health Organization) [27], was as follows: 7.8% underweight (<18.5 kg/m^2^), 66.0% normal weight (18.5–24.9 kg/m^2^), 17.0% overweight (25.0–29.9 kg/m^2^), 7.8% first degree obesity (30.0–34.9 kg/m^2^), 1.4% second degree obesity (35.0–39.9 kg/m^2^). Full characteristics of the respondents are summarised in Table 1.

In accordance with the Declaration of Helsinki, all subjects were informed about the purpose and method of the study and gave written consent to participate. The Bioethics Committee of the Silesian Medical University agreed to conduct the study (Resolution KNW/0022/KB1/47/12).

Exclusion criteria for the study were lack of nasal patency as demonstrated by a rhino-manometric test (total flow through the anterior nostrils less than 280 cm^3^), history of head trauma; chronic or acute nasal, sinus, or respiratory pathologies; history of COVID-19 infection; neurological disorders affecting olfaction; inability to understand the procedures during the study, and refusal to participate in the study. The exclusion of subjects from the study who did not have nasal patency was intended to eliminate such people in whom obstruction could cause short-term olfactory impairment. Pregnant women did not participate in the study.

Preparation of the volunteers for participation in the olfactory tests: Each participant was asked to avoid foods and spices with a strong taste and smell, e.g., garlic, the day before the test, to take special care with their personal hygiene and not to use cosmetics with a strong smell. The study was conducted after a 30 min acclimatisation to the olfactometric laboratory conditions. During this time, no food or drink (other than still water), chewing gum, smoking, applying cosmetics, or engaging in physical exertion was allowed.

### 2.2. Determination of the Olfactory Sensitivity Threshold

**Figure 1:**
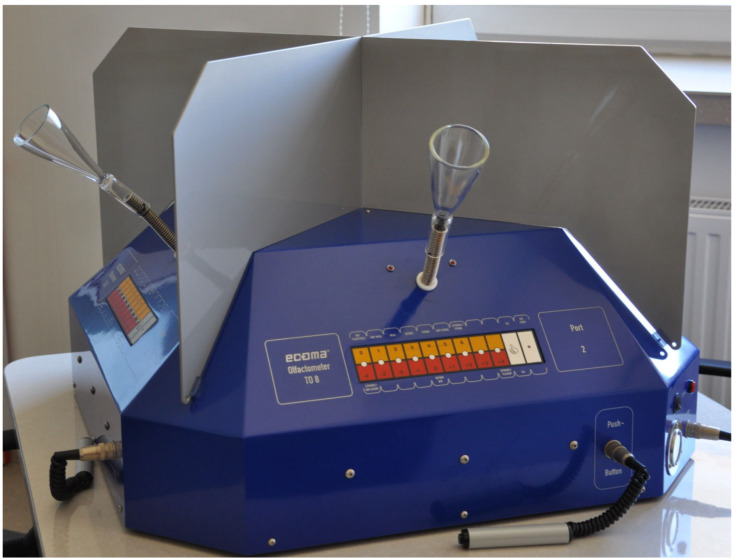
Measuring station in T08 olfactometer.

The olfactory sensitivity threshold to *n*-butanol was assessed by an ECOMA T08 olfactometer (ECOMA GmbH, Honigsee, Germany) (Figure 1) using the dynamic olfactometry method in accordance with the guidelines issued by the Polish Committee for Standardisation in accordance with European Standards PN-EN 13725:2007 [28]. The olfactometer diluted *n*-butanol at a concentration of 59.9 ppm with air and administered it to the participants’ stations in the following dilution steps 2^2^. *N*-butanol takes the following decreasing dilution factor values: from 65,636, 32,768, 16,384, 8192… to 32, 16, 8, 4, 0. Odour samples alternated with air were sent to the test subjects’ stations at a speed of 0.2 m/s, for 2.2 s. The participants’ task was to press a button when they smelled an odour other than air. The measuring cycle was stopped when the odour substance was correctly indicated at least twice, and no error was recorded if the air sample was selected. Before the test, the participants did not know the type of substance used. The assessment of the olfactory sensitivity threshold was carried out twice, the lower of the sensitivity thresholds obtained was chosen as the final result. In the statistical analysis, the dilution factor value of the sample was directly included.

### 2.3. Identification Test of Smell

**Figure 2:**
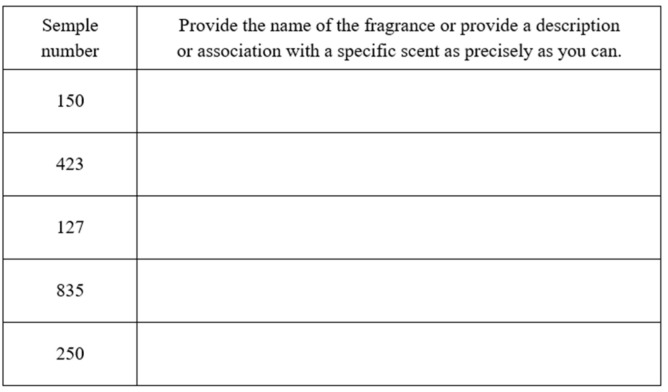
Answer sheet for the identification test of smell.

After a 15 min break, the participants moved to a separate room where they were asked to judge which odour they could smell, based on the smell of a substance which had been applied to a smelling strip. One odorant substance was applied to each smelling strip separately over a length of approximately 2 cm. The odorant substances were limonene (150), menthol (423), phenethyl alcohol (127), eugenol (835), and *n*-butanol (250) (Figure 2). Limonene smells like citrus, menthol smells like mint, phenethyl alcohol smells like flowers, eugenolsmells like cloves, and *n*-butanolsmells like an alcoholic chemical. Before the test, the participants were not familiarised with the above-mentioned odorant substances. The respondents described the names of the odours, as accurately as they could, with no further prompting; names similar to those presented above were accepted, e.g., limonene-lemon, lime, orange, citrus, lemonade. The outcome of the trial was the number of correctly recognised odours. When all five substance odours were correctly identified, the participant received 5 points.

### 2.4. Food Preference Test

**Figure 3:**
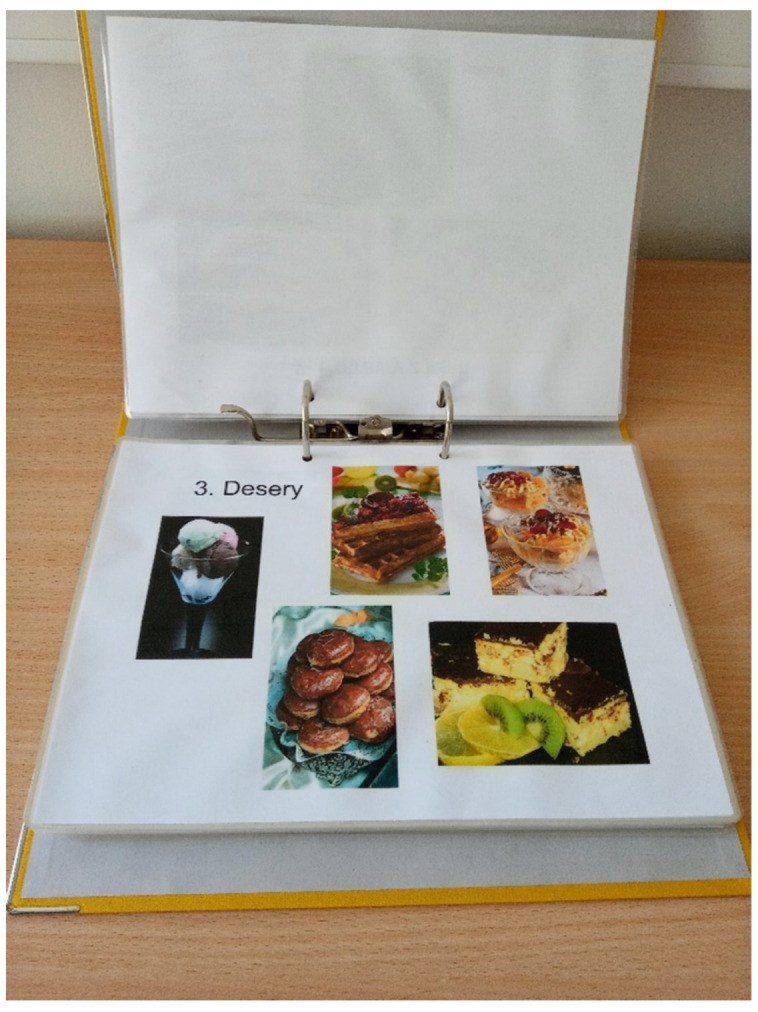
Photo album with pictures of twenty-four types of food and sugary carbonated drinks.

The food preference test was conducted prior to the olfactory test during the acclimatisation to the olfactometric laboratory conditions. The volunteers viewed a photo album with pictures of twenty-four types of food and sugary carbonated drinks (Figure 3).

**Figure 4:**
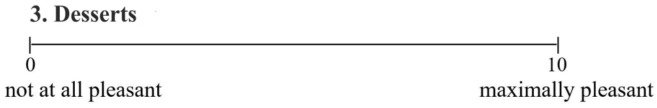
Linear scales for food preferences.

They were asked to state how pleasant they found the food they were looking at. They marked their answer on 10 cm linear scales labelled at one end ‘0—not at all pleasant’, and at the other end ‘10—maximally pleasant’ (Figure 4).

The score was the distance from zero to the point marked by the subject. The types of food assessed were fish dishes, egg dishes, sweet desserts, chocolate, sweets and jellybeans, crisps, dumplings, pasta, milk soup (this is a sweet dish made by pouring hot milk over things such as: boiled rice, pasta, oatmeal, chocolate chips or corn flakes, etc.), milk drinks, cheese, vegetables and salads, fruit, sausages and ham, beef and pork, poultry, bread, fast food, salty products, sour products, broth, soups, spicy dishes, seafood and sugary carbonated drinks.

### 2.5. Statistical Analysis

Statistical analysis was carried out using SPSS 21 software. A descriptive analysis was performed, then KMO (Kaiser–Meyer–Olkin) indices were checked for all the tested food. It is a test of sampling adequacy used in factor analysis. This helps determine whether the correlations between variables are suitable for factor extraction. Values > 0.80 are considered acceptable [29]. Next, Bartlett’s test was performed to examine whether the correlation matrix of variables is significantly different from an identity (uncorrelated) matrix. After this, a factor analysis of the main components was carried out using VARIMAX rotation—it gives a factor solution consisting of uncorrelated factors. After seven factors were identified, Factor Three was named ‘Sweet Products’ for the purposes of further analysis in the research. For linear multiple regression, an a priori power analysis was performed in order to determine the sample size necessary to detect small, medium, and large effects, in the case of six predictors. The analyses were performed by means of the software G*Power 3.1.9.6 [30,31]. Two-tailed analyses were performed; desired power of 95% was assumed. The sample size was determined for effect sizes (Cohen ƒ): 0.14, 0.39, and 0.59, which correspond to ƒ^2^, required by G*Power: 0.02, 0.15, and 0.35. The sample sizes required to detect small, medium, and large effects were 652, 89, and 40, respectively. Given the available resources, a sample of 283 participants was assumed, which assures excellent power to detect large and medium effects, but not small ones. Regression models were built for the entire ‘Sweet Products’ group and for each component independently, i.e., desserts, chocolate products, sweets and jellybeans, and bread. Among the sweet products included in the analysis in this study, and included in the statistical grouping, were sweet carbonated drinks (Factor One) and fruit (Factor Four). The predictors in the regression models were sex, age, BMI, pack-year, olfactory sensitivity and odour identification. Non-standardised regression coefficients (B) were given for numerical and dichotomous predictors, along with 95% confidence intervals. A coefficient of multiple determination (R^2^) value was also provided for each analysis, in addition to effect-size ratios for each predictor; these were expressed as eta-square. In the case of smoking, the indicator of the ‘pack-years’ addiction (number of cigarette packs smoked per day times years of smoking) was used as a predictor.

## 3. Results

### 3.1. Categorization of Dishes into Coherent Groups Based on the Principal Component Method with VARIMAX Rotation

A total of 25 types of food were analysed in order to assess the preferences, so a factor analysis was performed using the principal component method with VARIMAX rotation. This technique allows for a larger number of variables to be categorised into certain groups, such that the variables within each group relate to a similar factor. The KMO (Kaiser–Meyer–Olkin) value was 0.80, so it was acceptable [32]. Thanks to Bartlett’s test of sphericity, the hypothesis that the individual items are uncorrelated and that there was no factor structure among them (chi2 = 2408.64, *df* = 300, *p* < 0.001) was rejected. Only factors for which the eigen-value exceeded one were included. A clear factor solution was obtained. Overall, the seven factors identified explained a total of 62.10% of the variance for the scale items (Table 2).

**Figure 5:**
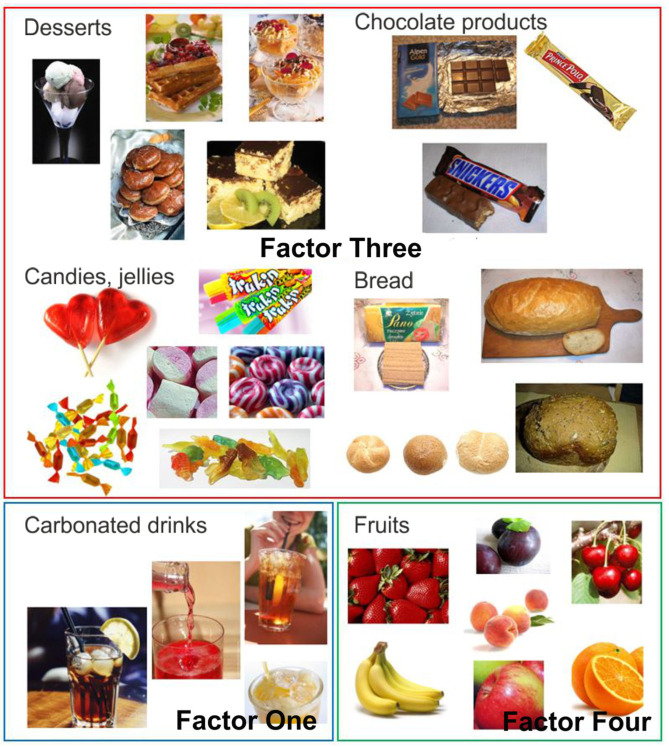
Photographs of food products, grouped according to factor analysis: Factor One—carbonated drinks; Factor Three—sweet products (desserts, chocolate products, candies, jellies and bread); Factor Four—fruits.

Factor Three had an eigenvalue of 2.69 and explained 10.78% of the variance value. The factor loadings after VARIMAX rotation for Factor Three, named in this study as ‘Sweet Products’, were desserts with a value of 0.85, chocolate products 0.84, sweets and jellies 0.77, and bread 0.35. The foods that comprised Factor Three ‘Sweet Products’, together with sweet carbonated drinks (Factor One) and fruit (Factor Four), are shown in Figure 5.

#### Ranking of Dishes

The values of the declared pleasure of eating foods are presented in Table 3.

### 3.2. Regression Model for the Factor Three ‘Sweet Products’ Group Including Variables Such as Sex, Age, BMI, Pack-Years, Olfactory Sensitivity Threshold, and Identification Test of Smell

**Figure 6:**
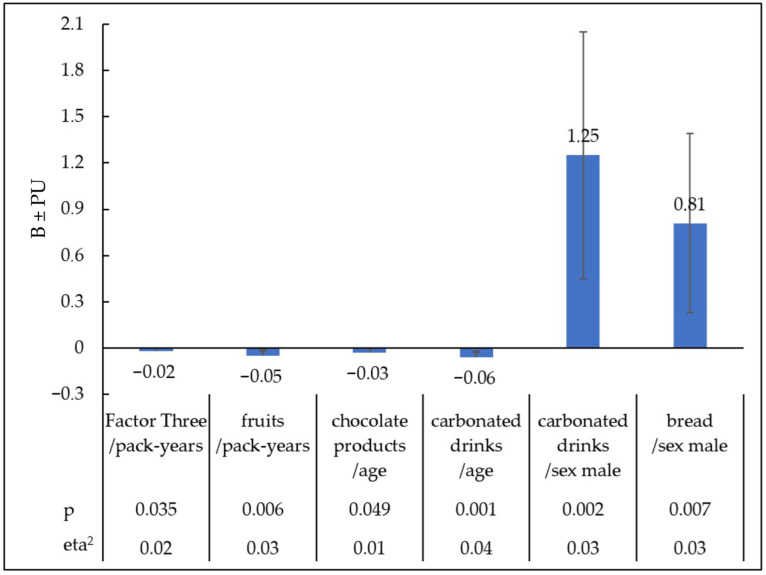
Statistically significant relationships between preferences for sweet products selected from different regression models and pack-years, age, and sex. B—Unstandardised regression coefficients; PU—confidence interval; eta^2^-effect size; *p*—level of significance.

Factor Three ‘Sweet Products’ became the dependent variable in the regression model (Table 4) (Figure 6).

The value of the multiple coefficient of determination in this model is R^2^_c_ = 0.07. Pack-years had the greatest impact on the increased preference for Factor Three. Greater tobacco addiction, as measured in pack-years, was associated with a declared reduction in the enjoyment of products in the category Factor Three ‘Sweet Products’ (B = −0.02; PU = −0.04, <0.01; t = −2.11, eta^2^ = 0.02; *p* = 0.035). Other predictors did not have a statistically significant effect on Factor Three ‘Sweet Products’.

### 3.3. Regression Models for Individual Sweet Products Including Variables Such as Sex, Age, BMI, Pack-Years, Olfactory Sensitivity Threshold, and Identification Test of Smell

From the analysis of the influence of factors on the particular type of food categorised as ‘Sweet Products’, including sweet carbonated drinks and fruit, a statistically significant effect of age, sex and the intensity of tobacco addiction was observed with some products (Table 5) (Figure 6).

With age, less pleasure was reported for chocolate products (B = −0.03; PU = −0.06, <0.01; t = −1.97, eta^2^ = 0.01; *p* < 0.049) and sweet carbonated drinks (B = −0.06; PU = −0.09, −0.02; t = −3.38, eta^2^ = 0.04; *p* = 0.001). The male sex increased the declared pleasure of consuming carbonated drinks (B = 1.25; PU = 0.45, 2.05; t = 3.07, eta^2^ = 0.03; *p* = 0.002), and bread (B = 0.81; PU = 0.23, 1.39; t = 2.74, eta^2^ = 0.03; *p* = 0.007). People with a higher intensity of tobacco addiction expressed in pack-years reported less pleasure from eating fruit (B = −0.05; PU = −0.08, −0.01; t = −2.78, eta^2^ = 0.03; *p* = 0.006).

## 4. Discussion

This study identified key factors influencing preferences for sweet-tasting foods among Polish adults. Of the 25 food products analysed, fruit and desserts elicited the highest pleasure ratings (mean scores: 8.63 and 8.24, respectively). Factor analysis revealed that desserts, chocolate, sweets, jellybeans, and bread formed a distinct group (Factor Three: “Sweet Products”), while fruit clustered with vegetables, salads, and cheese. The most significant predictor of reduced preference for Factor Three products was tobacco smoking severity. Notably, neither olfactory efficiency nor BMI significantly influenced preferences for any sweet products examined, suggesting that sensory mechanisms underlying sweet food preferences may be more complex than previously assumed.

### 4.1. Ranking of Preferences for Sweet-Tasting Food Products

Of the 25 types of food presented in this study, fruit and desserts were the products that were enjoyed the most. On a 10-point scale, the respective mean values for these parameters were 8.63 and 8.24, and the respective median values were 9.30 and 9.50. This finding is supported by previous research, which suggests that sweet tastes are generally considered beneficial, pleasant and safe, in contrast to bitter tastes, which can signal the presence of potentially poisonous substances [33,34]. A review by Hoffman et al. [21] concerning taste preferences showed that, in studies of eating habits and preferences, conducted before umami was officially recognised as a taste, sweet taste was the most popular in all analysed age groups. The search for sweet-tasting food is an innate behaviour in many mammalian species. The prevailing view is that sweet taste evolved as a way of detecting readily available sources of glucose, the primary energy substrate for the brain [35].

The high pleasure ratings observed for desserts and fruit in this study likely reflect the neurobiological basis of sweet taste preference. Sweet taste perception activates the brain’s reward system—a network of structures that evolved to reinforce beneficial behaviours. Dopamine, the key neurotransmitter in this system, mediates the subjective feeling of pleasure, thereby increasing motivation to consume sweet products [36,37,38,39]. This mechanism may explain why desserts consistently ranked among the most pleasurable foods in our cohort. The reward system’s response to sweet taste has been documented across age groups, with observations showing that sweet foods produce analgesic and calming effects in young children, possibly through the release of endogenous opioids from the central nervous system [36,37,38,39]. Understanding these neurobiological mechanisms helps contextualise our finding that sweet products form distinct preference clusters independent of their nutritional composition.

In this study, participants reported an average pleasure score of 6.24 when consuming sweets and jellybeans, which was significantly lower than for desserts or chocolate products (average: 7.61; median: 8.80). This observation is confirmed by the work of Markus et al. [40], who studied symptoms of food addiction (according to YFAS) in relation to different food categories. Where symptoms of food addiction were observed, they mainly concerned products with a high fat content combined with a sweet or salty taste and did not relate to products containing only large amounts of sugar. Furthermore, only the tendency to consume fatty foods was significantly correlated with an increased BMI. These findings support our observations, suggesting that it is not sugar alone, but overall energy density and individual susceptibility to the hedonic dimension of food that play a more important role in the development of food addiction and excessive body weight.

In this study, fruit was the most popular product group among the respondents. This is possibly due to its taste, nutritional value and ease of availability. In a previous study [41], increasing the proportion of fruit in a dessert was found to significantly improve its palatability compared to desserts with classic ingredient proportions, in which fruit was only an addition.

### 4.2. Food Groupings

The VARIMAX rotation used in this study revealed different patterns of high-carbohydrate food preference within the group. The coherent group, Factor Three ‘Sweet Products’, comprised desserts, chocolate, sweets and jellybeans. Bread was also included in this group, but its factor loading of only 0.35 indicates a weak affiliation. Although starch, the main ingredient in bread, is composed of glucose molecules, it does not produce a sweet taste because its complex structure does not interact with the T1R2 + T1R3 receptor. As the intensity of sweet taste perception is not proportional to the actual energy value of food, sensory mechanisms capable of detecting fats or polysaccharides, such as starch, may be more important from the perspective of effectively detecting energy sources [33,42]. The existence of a specialised starch receptor seems likely, as under the conditions of an experimental sweet taste receptor blockade with lactisole, study participants still perceived the characteristic taste of starch. People of Asian descent described it as being similar to the taste of rice, while European participants said it was similar to the taste of bread [43]. The hypothesis that there is a distinct starch taste can be questioned, as the amylase in saliva quickly breaks starch down into maltose and other substances, which activate sweet taste receptors. This may explain why bread belongs to the Factor Three group. Common characteristics of the Factor Three group include high energy value resulting from high carbohydrate content, mainly sucrose, and, in the case of chocolate products, desserts (and fats), a significant degree of processing and low nutritional value (with the exception of wholemeal bread). This grouping revealed that, despite their sweet taste, fruit preferences were not aligned with those of the Factor Three group, but were more consistent with preferences for vegetables, salads, cheese and spicy dishes. This phenomenon can be explained by the fact that, in nutritional recommendations, fruit and vegetables are often treated as a single food category. This is because they are both plant-based and highly nutritious. Both product groups are rich in fibre, vitamins, minerals, and antioxidants. Combining them in meals and snacks promotes better nutrition and helps to prevent many diseases, making it easier to follow health recommendations such as ‘eat five portions of fruit and vegetables a day’ [44,45].

The preference for sweet, carbonated drinks was different to that for the Factor Three ‘Sweet products’, but it was consistent with Factor One, i.e., unhealthy food, as described in the study by Hartman-Petrycka et al. [25]. This observation has been confirmed by studies which have found an association between increased consumption of fast food and increased consumption of non-alcoholic carbonated drinks, usually with a sweet taste [46,47], as well as a decrease in the consumption of fruit and vegetables [48].

### 4.3. Factors Influencing Preferences for the Factor Three ‘Sweet Products’ Group as a Whole, and for Individual Types of High-Carbohydrate Food

Our study examined the potential influence of various predictors on the preferences of the Factor Three ‘Sweet Products’ group, as well as on the preferences for individual food products analysed within this group and for fruit and sweetened beverages. The predictors considered were age, sex, BMI, tobacco addiction and olfactory sensitivity, as measured by two types of tests: *n*-butanol dilution step and odour identification. Of all the predictors included in the study, the severity of tobacco addiction had the most significant impact on the preferences of the Factor Three “Sweet Products” group. The more severe the addiction, the weaker the preference for Factor Three products.

Nicotine is an alkaloid found in tobacco leaves that induces the release of dopamine by binding to nicotinic cholinergic receptors (nAChRs) in the brain. Similar to sweet-tasting foods, nicotine activates the reward system, which is responsible for feelings of pleasure and satisfaction [49,50]. Studies using functional magnetic resonance imaging (fMRI) have shown that, in individuals dependent on nicotine, activation of the ventral striatum (a key component of the reward system) in response to stimuli other than tobacco (e.g., tasty food or monetary rewards) is impaired [51]. These results indicate that in nicotine-dependent individuals, dopaminergic activation of the reward system occurs primarily in response to nicotine, which may result in a weakened responsiveness of this system to natural rewards. This could explain why smokers report lower satisfaction from meals and are more likely to reach for a cigarette afterwards [52]. This fact may partially explain the results of this study, where sweet flavours appeared to be less satisfying for cigarette smokers.

Smoking and appetite regulation are closely related. In a study by Mineur et al. [53] nicotine reduced appetite and limited food intake by activating pro-opiomelanocortin (POMC) neurons [53].

The results of this study can be explained by previous research showing that women smokers have reduced sensitivity to sweet tastes compared to non-smoking women, as evidenced by a higher sucrose detection threshold. It was also found that sensitivity to sucrose decreased as the severity of addiction, measured in pack-years, increased [54]. Reduced taste perception in smokers was also reported by Chéruel et al. [55], who additionally found that it can take from several weeks to several months for taste perception to return to normal after quitting smoking. Tomassini et al. [56] conducting observations on animal models, discovered that prolonged exposure to nicotine leads to morphological changes in the fungiform papillae, which may impair the functioning of taste buds. Although Perkins et al. [57] did not observe significant differences in the perception of sweet taste between smokers and non-smokers, they noted that smokers gave a sweet taste a lower hedonic rating, which may influence their food preferences. The relationship between smoking and olfactory function appears complex and may depend on smoking status. While Da Ré et al. [22] demonstrated in a systematic review that smoking impairs olfactory function, a comprehensive meta-analysis by Ajmani et al. [58] found that this association was significant only for current smokers (OR 1.59, 95% CI 1.37–1.85), but not for former smokers (OR 1.05, 95% CI 0.91–1.21), suggesting potential reversibility of smoking-related olfactory effects. Additionally, several population-based studies have reported no significant association between smoking status and olfactory performance [59,60,61]. This inconsistency in the literature may explain why our study found no significant relationship between olfactory efficiency and preference for sweet products. The lack of association observed in our data suggests that the effect of smoking on taste preferences may operate through alternative pathways beyond olfactory impairment, or that smoking-related changes in olfaction may not be sufficiently severe to impact hedonic responses to sweet foods.

Given the close relationship between smell and taste, this also contributes to a weakened perception of taste. The sense of smell acts as a biological stimulus that initiates food intake and plays a key role in the initial phase of digestion. However, the results of our study suggest that its influence on preferences for sweet foods may be smaller than previously thought. Analysis showed no statistically significant relationship between olfactory sensitivity and preference for any of the high-carbohydrate products tested. This may be because desserts are usually served cold (e.g., ice cream), while other sweets are served at room temperature, which limits their aroma. Low temperatures inhibit the release of volatile aroma compounds and reduce the effectiveness of taste receptors, suppressing the perception of aroma and taste [23].

Factor analysis of each sweet food item revealed that preferences for desserts, sweets, and jellybeans were not significantly related to any of the analysed factors. However, preferences for chocolate products were influenced by the age of the respondents. These products were more popular among younger people, though the statistical significance of this effect was low (*p* = 0.049). It is well documented that preference for sweet tastes decreases with age. Children and adolescents have a preference for higher sucrose concentrations, whereas older adults have reduced sensitivity to sweet tastes, which may contribute to a lower preference for sweet foods [21]. While overall sweet taste preference decreases with age [21], research suggests that olfactory impairment in older adults may influence their chocolate preferences [62].

Bread was more popular among men than women (*p* = 0.007). This finding is consistent with that of Hartman-Petrycka et al. [26], who found that men show a greater preference for animal products, including cold cuts, than women. On the other hand, women are more interested in healthy eating and weight control, which may explain their lower preference for bread [63]. Importantly, women are much more likely than men to suffer from gluten intolerance in the form of coeliac disease or non-coeliac gluten sensitivity (NCGS). Meta-analyses show that women have a 40% higher risk of coeliac disease and an 80–90% higher risk of NCGS than men. This difference may be due to women’s greater susceptibility to autoimmune diseases, as well as their tendency to report symptoms and seek diagnosis more frequently [64,65].

As discussed in detail in the publication by Hartman-Petrycka et al. [25] on preferences for unhealthy fast food, sweet, carbonated drinks were more popular among younger people and men. Preferences for fruit depended on the severity of nicotine addiction and were weaker the stronger the addiction to tobacco. Research findings indicate that smokers consume fewer portions of fruit per day on average than non-smokers [66,67,68]. In a study conducted by Miyoshi et al. [66], participants reported that eating foods rich in fat was associated with a greater likelihood of craving a cigarette. On the other hand, foods with a sweet or sour taste, such as fruit, dairy products, vinegar and sugar, which are low in fat, were included in the group of products that reduced the likelihood of developing such cravings. The organic acids contained in this group of foods, such as citric, malic, lactic and acetic acids, can lower the pH of saliva, which potentially reduces nicotine absorption. This mechanism may partly explain why smokers avoid these products.

### 4.4. Limitations

Although a relatively large group of participants took part in the study, it was not representative of Poland’s general population. As outlined in the methodology, the group was dominated by medical students, which impacted the demographic composition. A higher percentage of women, a lower average age, a smaller proportion of overweight and obese individuals, and a greater preference for healthier products were observed. This may be attributed to the group’s enhanced nutritional knowledge. The study aimed to analyse the relationship between the demographic variables characterising the participants and their food preferences, rather than identifying the most frequently chosen food products in the Polish population (Table 2). No dietary interviews were conducted; preferences were assessed based on the declared pleasure of consuming the selected product groups. However, it should be remembered that declarations do not directly reflect actual consumption.

Self-assessment of preferences may be subject to social desirability bias, whereby respondents adjust their answers to align with social norms and expectations. This significantly limits the reliability of studies based on declarations by respondents, particularly in the context of eating behaviours, where social norms are deeply rooted. Research by Barros et al. [69] shows that women tend to underestimate their consumption of products considered unhealthy, such as white bread and beer, while consumption of healthy foods such as fruit and vegetables is reported at higher levels by both sexes [69]. Knox et al. [70] showed that social disapproval of sweetened beverages may lead to lower reported consumption compared to that reported by online respondents (a difference of 0.63 beverages per week). Similarly, children studied by Clemente et al. [71] reported a lower consumption of sweets and desserts than they actually consumed. Conversely, Cerri et al. [72] showed that consumers overestimated their consumption of organic fruit. To limit the impact of these errors, the questionnaire focused solely on the subjective assessment of the pleasure associated with consuming individual products rather than on how frequently they were consumed. The surveys were anonymous and completed independently to minimise the influence of social pressure associated with the presence of the data collector.

Several additional limitations warrant consideration. One limitation of the statistical analysis is that the predictors studied were very heterogeneous among themselves (e.g., sex—a dichotomous variable; olfactory sensitivity—a continuous test variable). Therefore, the results should be treated with some caution. As the research was exploratory, no corrections for multiple comparisons were performed, which may limit the value of the results.

Additionally, olfactory function was assessed using subjective self-report measures (*n*-butanol dilution and odour identification tests) rather than objective neurophysiological methods, which may have limited our ability to detect subtle sensory differences. The cross-sectional design precludes causal inference regarding the relationships between tobacco use, olfactory function, and food preferences. Longitudinal studies tracking changes in taste preferences following smoking cessation would provide stronger evidence or causal mechanisms. Furthermore, we did not assess the duration or intensity of tobacco exposure beyond addiction severity, variables that may differentially impact sensory function. Finally, the absence of biochemical verification of smoking status (e.g., cotinine levels) may have resulted in misclassification of exposure.

### 4.5. Future Research Directions

Future studies should employ objective measures of olfactory and gustatory function, such as electrophysiological recordings or functional neuroimaging, to elucidate the neural mechanisms underlying the relationship between sensory perception and food preferences. Longitudinal designs tracking individuals before and after smoking initiation or cessation would help establish temporal relationships and causality. Given the unexpected finding that olfactory efficiency did not predict sweet food preferences, research should investigate alternative sensory pathways, such as trigeminal chemosensation or oral somatosensation, that may contribute to hedonic responses to sweet foods. Additionally, studies incorporating biomarkers of metabolic function (e.g., glucose tolerance, insulin sensitivity) alongside sensory assessments would help clarify whether metabolic factors mediate the relationship between smoking and taste preferences. Finally, replication studies in more representative population samples, including older adults and individuals with clinical conditions affecting taste and smell (e.g., chronic rhinosinusitis, neurodegenerative diseases), are needed to assess the generalizability of these findings.

## 5. Conclusions

Of all the dishes analysed in the study, desserts and fruit provided the highest declared pleasure. Depending on the type of sweet food or drink, food preferences varied and were also dependent on other factors. In terms of declared pleasure, desserts, chocolate, sweets, jellybeans and bread formed a common group (Factor Three: ‘Sweet Products’), while sweet carbonated drinks belonged to Factor One (‘junk food’) and fruit belonged to Factor Four, alongside vegetables, salads, cheese and spicy dishes.

The most important factors influencing the preference for sweet foods and drinks were the severity of tobacco addiction, age and sex. No significant influence of olfactory efficiency or BMI on the preference for any of the sweet products evaluated in the study was found.

## Figures and Tables

**Table 1:** Characteristics of the study participants.

	N	Mean	Median	SD	Minimum	Maximum
Age (Years)	283	29.22	23.00	13.44	18.00	82.00
BMI	282	23.33	22.21	4.17	16.65	36.73
No. of years as a smoker	283	2.80	0.00	7.27	0.00	44.00
No. of cigarettes a day	283	2.27	0.00	5.91	0.00	50.00
Severity of addiction (pack-years)	283	1.83	0.00	6.86	0.00	60.00
Olfactory sensitivity threshold (serial dilution)	283	7021	1024	15,208	0.00	65,636
Identification of smell	283	3.94	4.00	1.08	0.00	5.00

**Table 2:** Results of VARIMAX rotation for all types of dishes. Items marked in grey were selected for further analysis.

Factors	Types of Dishes	Factor Loadings	Eigenvalue	% of Variance Explained
Factor One—‘junk food’	Crisps	0.76	2.92	11.66
	Salty snacks	0.76		
	Fast food	0.75		
	Carbonated drinks	0.61		
	Sour products	0.40		
Factor Two—‘meat, fish and seafood’	Beef, pork and veal	0.76	2.60	10.38
	Cured meats	0.73		
	Poultry	0.70		
	Fish dishes	0.53		
	Seafood	0.52		
Factor Three—‘sweet products’	Desserts	0.85	2.69	10.78
	Chocolate products	0.84		
	Candies and jellies	0.77		
	Bread	0.35		
Factor Four—‘vegetable and fruits, cheeses and spicy dishes’	Vegetables and salads	0.76	2.02	8.09
	Fruit	0.67		
	Cheeses	0.49		
	Spicy dishes	0.44		
Factor Five—‘flour-based and egg-based dishes’	Egg dishes	0.79	2.00	8.01
	Pasta	0.62		
	Flour-based dishes	0.61		
Factor Six—‘soups’	Broth	0.79	1.86	7.43
	Soups	0.77		
Factor Seven—‘milk products’	Milk soup	0.79	1.68	6.74
	Milk products	0.71		

**Table 3:** The values of the declared pleasure of eating various types of dishes, ranging from the most popular ones. Dishes marked in grey are the ones that have been identified by statistical analysis as a common ‘Factor Three’—‘sweet products’, and additionally carbonated drinks—Factor One and fruits—Factor Four.

	N	Mean	Median	SD	Minimum	Maximum
Fruit	282	8.63	9.30	1.82	1.00	10.00
Desserts	283	8.24	9.50	2.47	0.00	10.00
Vegetables and salads	282	7.84	8.50	2.26	0.50	10.00
Poultry	282	7.69	8.25	2.29	0.00	10.00
Chocolate products	283	7.61	8.80	2.84	0.00	10.00
Bread	282	7.38	7.80	2.18	0.20	10.00
Pasta	282	7.05	7.60	2.45	0.00	10.00
Egg dishes	283	6.90	7.30	2.61	0.00	10.00
Flour-based dishes	283	6.89	7.10	2.55	0.00	10.00
Soups	283	6.79	7.10	2.58	0.00	10.00
Broth	283	6.72	7.70	3.11	0.00	10.00
Cheeses	281	6.67	7.00	2.72	0.00	10.00
Cured meats	282	6.66	7.20	2.82	0.00	10.00
Fish dishes	283	6.66	7.00	2.67	0.00	10.00
Beef, pork and veal	282	6.54	7.20	3.01	0.00	10.00
Candies and jellies	282	6.24	6.90	3.20	0.00	10.00
Milk products	281	6.03	6.10	2.80	0.00	10.00
Sour products	282	6.00	6.00	2.88	0.00	10.00
Fast food	283	5.71	6.30	3.38	0.00	10.00
Crisps	283	5.58	6.00	3.16	0.00	10.00
Spicy dishes	283	5.47	5.50	3.30	0.00	10.00
Carbonated drinks	282	5.03	5.00	3.11	0.00	10.00
Salty snacks	283	4.84	5.00	2.93	0.00	10.00
Milk soup *	282	3.52	2.90	3.14	0.00	10.00
Seafood	283	3.38	2.10	3.42	0.00	10.00

* milk soup (this is a sweet dish made by pouring hot milk over things such as: boiled rice, pasta, oatmeal, chocolate chips or corn flakes, etc.).

**Table 4:** The effect of predictors such as sex, age, BMI, pack-years, the *n*-butanol dilution step, odour identification of the group of dishes selected in the factor analysis as Factor Three ‘sweet products’; B—Unstandardised regression coefficients; PU—confidence interval; R^2^_c_—multiple determination coefficient; eta^2^-effect size; t—*t* Statistic; *p*—level of significance.

Dependent Variables	R^2^_c_	Predictors	B	PU		t	eta^2^	*p*
Factor Three ‘sweet products’	0.07	Constant	0.69	−0.22	1.60	1.50	0.01	0.135
		Sex	−0.06	−0.32	0.21	−0.44	<0.01	0.662
		Age	<0.01	−0.02	0.01	−0.75	<0.01	0.453
		BMI	−0.03	−0.06	0.01	−1.57	0.01	0.118
		Pack-years	−0.02	−0.04	<0.01	−2.11	0.02	0.035
		Olfactory sensitivity threshold	<0.01	<0.01	<0.01	<0.01	<0.01	0.999
		Identification test of smell	0.04	−0.07	0.16	0.75	<0.01	0.454

**Table 5:** Influence of predictors such as sex, age, BMI, pack-years, *n*-butanol dilution step, odour identification on the declared pleasure of individual dishes identified in the factor analysis as Factor Three ‘sweet products’ and fruits and carbonated drinks; B—Unstandardised regression coefficients; PU—confidence interval; R^2^_c_—multiple determination coefficient; eta^2^-effect size; t—*t* Statistic; *p*—level of significance.

Dependent Variables	R^2^_c_	Predictors	B	PU		t	eta^2^	*p*
chocolate products	0.07	Constant	8.22	5.69	10.75	6.39	0.13	<0.001
		Sex	−0.39	−1.12	0.35	−1.04	<0.01	0.300
		Age	−0.03	−0.06	<0.01	−1.97	0.01	0.049
		BMI	0.01	−0.09	0.10	0.14	<0.01	0.887
		Pack-years	−0.04	−0.10	0.01	−1.58	0.01	0.116
		Olfactory sensitivity threshold	<0.01	<0.01	<0.01	0.27	<0.01	0.788
		Identification test of smell	0.19	−0.13	0.51	1.15	<0.01	0.252
candies and jellies	0.06	Constant	7.31	4.43	10.19	5 < 0.01	0.08	<0.001
		Sex	0.18	−0.66	1.01	0.42	<0.01	0.677
		Age	−0.02	−0.06	0.01	−1.20	0.01	0.231
		BMI	−0.07	−0.17	0.04	−1.25	0.01	0.214
		Pack-years	−0.04	−0.11	0.02	−1.44	0.01	0.150
		Olfactory sensitivity threshold	<0.01	<0.01	<0.01	−0.03	<0.01	0.977
		Identification test of smell	0.25	−0.12	0.62	1.34	0.01	0.180
desserts	0.07	Constant	9.05	6.81	11.29	7.95	0.19	<0.001
		Sex	−0.34	−0.99	0.32	−1.01	<0.01	0.312
		Age	−0.01	−0.04	0.02	−0.59	<0.01	0.553
		BMI	−0.03	−0.12	0.05	−0.77	<0.01	0.440
		Pack-years	−0.03	−0.08	0.02	−1.21	0.01	0.227
		Olfactory sensitivity threshold	<0.01	<0.01	<0.01	0.92	<0.01	0.358
		Identification test of smell	0.16	−0.13	0.45	1.10	<0.01	0.272
bread	0.08	Constant	6.75	4.77	8.74	6.70	0.14	<0.001
		Sex	0.81	0.23	1.39	2.74	0.03	0.007
		Age	0.02	<0.01	0.05	1.59	0.01	0.114
		BMI	−0.05	−0.13	0.02	−1.47	0.01	0.143
		Pack-years	−0.02	−0.06	0.02	−0.94	0.00	0.346
		Olfactory sensitivity threshold	<0.01	<0.01	<0.01	0.92	0.00	0.361
		Identification test of smell	0.06	−0.20	0.31	0.43	0.00	0.667
carbonated drinks	0.06	Constant	4.26	1.50	7.01	3.04	0.03	0.003
		Sex	1.25	0.45	2.05	3.07	0.03	0.002
		Age	−0.06	−0.09	−0.02	−3.38	0.04	0.001
		BMI	0.01	−0.10	0.11	0.10	<0.01	0.918
		Pack-years	0.05	−0.01	0.11	1.59	0.01	0.113
		Olfactory sensitivity threshold	<0.01	<0.01	<0.01	0.33	<0.01	0.745
		Identification test of smell	0.15	−0.20	0.50	0.85	<0.01	0.398
fruits	0.06	Constant	9.16	7.52	10.80	10.98	0.31	<0.001
		Sex	−0.30	−0.78	0.18	−1.24	0.01	0.216
		Age	0.01	−0.01	0.03	0.74	<0.01	0.458
		BMI	−0.03	−0.09	0.03	−0.94	<0.01	0.346
		Pack-years	−0.05	−0.08	−0.01	−2.78	0.03	0.006
		Olfactory sensitivity threshold	<0.01	<0.01	<0.01	−0.86	<0.01	0.391
		Identification test of smell	0.11	−0.09	0.32	1.08	<0.01	0.281

## Data Availability

The dataset supporting the findings of this study is openly available at https://doi.org/10.71804/ctaw-4172.

## Abbreviations

The following abbreviations are used in this manuscript:

BMI	Body Mass Index
ECOMA	European Committee for Olfactometry Manufacturers Association
FAO	Food and Agriculture Organization
WHO	World Health Organization
NCGS	Non-Celiac Gluten Sensitivity
POMC	Pro-opiomelanocortin
T1R2 + T1R3	Sweet Taste Receptor Complex (Type 1 Receptor Family)
VARIMAX	Orthogonal Rotation Method in Factor Analysis
YFAS	Yale Food Addiction Scale
